# Identifying Human Disease Genes through Cross-Species Gene Mapping of Evolutionary Conserved Processes

**DOI:** 10.1371/journal.pone.0018612

**Published:** 2011-05-04

**Authors:** Martin Poot, Alexandra Badea, Robert W. Williams, Martien J. Kas

**Affiliations:** 1 Department of Medical Genetics, University Medical Center Utrecht, Utrecht, The Netherlands; 2 Center for In Vivo Microscopy, Duke University Medical Center, Durham, North Carolina, United States of America; 3 Department of Anatomy and Neurobiology, University of Tennessee Health Science Center, Memphis, Tennessee, United States of America; 4 Department of Neuroscience and Pharmacology, Rudolf Magnus Institute of Neuroscience, University Medical Center Utrecht, Utrecht, The Netherlands; Chiba University Center for Forensic Mental Health, Japan

## Abstract

**Background:**

Understanding complex networks that modulate development in humans is hampered by genetic and phenotypic heterogeneity within and between populations. Here we present a method that exploits natural variation in highly diverse mouse genetic reference panels in which genetic and environmental factors can be tightly controlled. The aim of our study is to test a cross-species genetic mapping strategy, which compares data of gene mapping in human patients with functional data obtained by QTL mapping in recombinant inbred mouse strains in order to prioritize human disease candidate genes.

**Methodology:**

We exploit evolutionary conservation of developmental phenotypes to discover gene variants that influence brain development in humans. We studied corpus callosum volume in a recombinant inbred mouse panel (C57BL/6J×DBA/2J, BXD strains) using high-field strength MRI technology. We aligned mouse mapping results for this neuro-anatomical phenotype with genetic data from patients with abnormal corpus callosum (ACC) development.

**Principal Findings:**

From the 61 syndromes which involve an ACC, 51 human candidate genes have been identified. Through interval mapping, we identified a single significant QTL on mouse chromosome 7 for corpus callosum volume with a QTL peak located between 25.5 and 26.7 Mb. Comparing the genes in this mouse QTL region with those associated with human syndromes (involving ACC) and those covered by copy number variations (CNV) yielded a single overlap, namely *HNRPU* in humans and *Hnrpul1* in mice. Further analysis of corpus callosum volume in BXD strains revealed that the corpus callosum was significantly larger in BXD mice with a *B* genotype at the *Hnrpul1* locus than in BXD mice with a *D* genotype at *Hnrpul1* (F = 22.48, p<9.87*10^−5^).

**Conclusion:**

This approach that exploits highly diverse mouse strains provides an efficient and effective translational bridge to study the etiology of human developmental disorders, such as autism and schizophrenia.

## Introduction

The corpus callosum is the fibrous structure that connects both hemispheres of the cortex in all placental mammals [1], [2]. In humans, this bridge is made up of more than 100 million axons of neocortical neurons routing information between the left and the right sides of the brain [3], [4]. Improper development of the corpus callosum may manifest itself in infancy by feeding problems, delays in acquiring proper posture and the ability to walk, and impairments in hand-eye coordination, speech, and visual and auditory memory. In mild cases, symptoms such as repetitive speech, social awkwardness, rigid thinking, poor problem solving, and odd communication patterns may appear during elementary school years. During puberty, children with an abnormal corpus callosum (ACC) often fall behind in social understanding, social communication, comprehension of non-literal language, problem solving, executive skills, recognition of emotions, self-awareness, and personal insight. Given the lack of specificity of symptoms of an ACC, it is critical to properly diagnose an ACC, which may either occur as an isolated clinical entity, as part of a syndrome (for reviews see: [1], [2], [5], [6]) or in association with complex phenotypes such as autism or pontocerebellar hypoplasia [7], [8]. Recent studies have put forward potential candidate genetic mechanisms underlying an ACC in humans, however, the question remains how these genes contribute to abnormal development of this brain region relevant to proper human functioning.

For example, developmental processes in mammals are presumed to be controlled by complex genetic networks often involving interaction of several loci and genes [9], [10]. Unraveling the myriad interactions within these networks is a major task in clinical genetics, which faces the challenge of correctly interpreting the phenotypic manifestations of perturbed development in relation to data on genome alterations. Since these fundamental developmental processes are among the evolutionary most conserved, we hypothesize that the underlying genetic networks and their interaction patterns are also highly conserved. Here, we present an approach to interpret human genomic data relating to perturbed developmental disease processes by making use of their evolutionary conservation through natural genetic variation within and across species. As a test case, we have analyzed a locus involved in the development of the corpus callosum in the human and the mouse brain.

## Results

### Prioritizing candidate loci and genes

Following a procedure used in a recent study of patients with multiple congenital anomalies and mental retardation [11] we used a disease cohort-specific compilation of genes involved in syndromes involving an ACC to prioritize contributing loci, genes, and biological processes. For the 61 autosomal recessive and dominant, X-linked, metabolic, and chromosomal syndromes which involve an ACC, 51 human candidate genes have been identified (**Table 1**). Of these 19 (*ARX*, *ATRX*, *DCX*, *EFNB1*, *EP300*, *FCMD*, *FGFR1*, *FLNA*, *GLI3*, *HESX1*, *L1CAM*, *LARGE*, *MID1*, *PAFAH1B1*, *PAX6*, *PTCH*, *RELN*, *WHSC1*, and *ZFHX1B*) fit into the Gene Ontology (GO) category of development (GO:0007275; p value<0.0001 and Bayes Factor 15). The more narrow category of neurogenesis (GO:0007399) includes 11 genes (*DCX*, *EFNB1*, *EP300*, *FCMD*, *FLNA*, *HESX1*, *L1CAM*, *LARGE*, *PAFAH1B1*, *PAX6*, and *ZFHX1B*; p<0.0001 and Bayes Factor 16). Both the p values (assuming a normal distribution of likelihoods) and the Bayes factor, indicating the fold-likelihood that a model fits the data vs. the neutral null-hypothesis, indicate a highly significant association of these GO categories with an ACC [12]. Accordingly, an ACC may result from defective functioning of any or several genes from a relatively limited set of genes required for proper neurodevelopment.

**Table 1:** Syndromes involving an abnormal corpus callosum (ACC).

*Syndrome*	*Locus*	*Gene*	*OMIM*	*Syndrome*	*Locus*	*Gene*	*OMIM*
**Autosomal-dominant**							
Apert	10q26	FGFR2	101200	Lissencephaly 3	12q12-q14	TUBA1A	611603
Basal cell nevus	9q22.3	PTCH	109400	Rubinstein-Taybi	16p13.3	CREBBP	180849
					22q13	EP300	180849
Greig cephalo-polysyndactyly	7p13	GLI3	175700	Septo-Optic dysplasia (SOD)	3p21.2-p21.1	HESX1	182230
Kallmann 2	8p11.2-p11.1	FGFR1	147950	Sotos	5q35	NSD1	117550
**Autosomal-recessive**							
Acrocallosal	7p13	GLI3	200990	Lissencephaly 2	7q22	RELN	257320
Andermann	15q13-q14	SLC12A6	218000	Marden-Walker			248700
Aniridia type II	11p13	PAX6	106210	Meckel-Gruber	17q22-q23		249000
Coffin-Siris	7q32-q34		135900	Microcephalic osteodysplastic primordial dwarfism (MOPD) type 1			210710
Dincsoy			601016				
Fryns			229850				
Fukuyama congenital muscular dystrophy	9q31	FCMD	253800	MOPD type 3			210730
Hydrolethalus	11q24.2	HYLS1	236680	Mowat-Wilson	2q22	ZFHX1B	235730
Joubert	9q34.3		213300	Muscle-eye-brain disease	1p34-p33	POMGNT1	253280
	11p12-q13.3		608091	Neu-Laxova			256520
	8q21.13-q22.1	TMEM67	610688	Septooptic dysplasia	3p21.2-p21.1	HESX1	182230
	6q23.3	AHI1	608629	Toriello-Carey			217980
	2q13	NPHP1	609583	Vici			242840
	3q11.2	ARL13B	612291	Walker-Warburg	9q34.1	POMT1	607423
	4p15.3	CC2D2A	612285		14q24.3	POMT2	607439
	16q12,2	RPGRIP1L	611560		19q13.3	FKRP	606596
	12q21.3	CEP290	610188		22q12.3-q13.1	LARGE	603590
Lowry-Wood			226960		9q31	FKTN	607440
Lyon			225740	Warburg-Micro	2q21.3	RAB3GAP	600118
**X-linked**							
Aicardi	Xp22		304050	X-linked lissencephaly	Xq22.3-q23	DCX	300067
ATR-X	Xq13	ATRX	301040	Lissencephaly X-linked 2	Xp22.13	ARX	300215
Aqueductal stenosis/ hydrocephalus (MASA syndrome; X linked) or Hydrocephalus due to congenital stenosis of aqueduct of Sylvius	Xq28	L1CAM	307000	Lujan-Fryns	Xq13		309520
				Microphthalmia with linear skin defects	Xp22.31		309801
				Opitz	Xp22	MID1	300000
				Opitz-Kaveggia	Xq13	MID12	305450
				Oro-facial digital type 1	Xp22.3-p22.2	CXORF5	311200
Craniofrontonasal	Xq12	EFNB1	304110	Periventricular heterotopia	Xq28		300049
Lenz micropthalmia	Xq27-q28		309800	Proud	Xp22.13	ARX	300004
**Metabolic disorders**							
Fumarase deficiency	1q42.1	FH	606812	Smith-Lemli-Opitz	11q12-q13	DHCR7	270400
Glycine encephalopathy	9p22	GCSP	606812				
PDH deficiency	Xp22	PDHA1	312170	Zellweger	6q23-q24	PEX3	214100
**Chromosomal (contiguous gene and deletion) syndromes**							
ACC with ectodermal dysplasia (hypohidrotic)			225040	Miller Dieker Lissenecephaly	17p13.3		247200
Delleman syndrome (Oculocerebrocutaneous)			164180	Ocular motor apraxia (Cogan-syndrome)	2q13		257550
Lissencephaly type I	17p13.3	LIS1	607432	Opitz GBBB	22q11.2		145410
Lissencephaly type III	17p13	PAFAH1B1	601545	Wolf–Hirschhorn	4p16.3	WHSC1	194190

From recent studies of structural genome rearrangements, 15 loci and consensus regions have emerged [13]–[25]. Results from several studies converge onto regions 1q44, 6q27, and 15q21.2. One could even interpret these studies as describing shared consensus regions within bands 1q44, 6q27 and 15q21.2. The studies of 1q44 deletions, however, have thus far resulted in three mutually exclusive consensus regions [13]–[15], [26], [27]. The initially proposed candidate gene *AKT3* has subsequently been disputed [14], [15], [28]. Submitting the candidate genes indicated by these studies of structural genome rearrangements to an analysis of shared Gene Ontologies did not yield a similar signature biological process as found with syndromal genes (see above). This is not too surprising since some of the CNVs are relatively large and often contain “bystander” genes that are not likely to be involved in a patient's phenotype. To overcome this drawback, we sought a novel approach by reasoning that the evolutionary conservation of the development of the corpus callosum among mammals may provide us with a clue.

### Gene mapping with crosses of inbred mouse strains

Unbiased phenotype-driven approaches in mice may also contribute to identification of genetic loci relevant to the development of the corpus callosum in humans. Analyses of mouse genetic reference populations (GRPs) allow for systematic identification of quantitative trait loci (QTL) using controlled genetic background and environmental conditions. For instance, a recombinant inbred (RI) panel which is generated from a cross between two inbred strains [29], [30] (e.g., C57BL/6J×DBA/2J (BXD)) [31]; followed by an F1 intercross and 20 generations of inbreeding has proven to be a powerful instrument for studies of complex genetic traits on the basis of natural variation [29]–[31].

**Figure 1:**
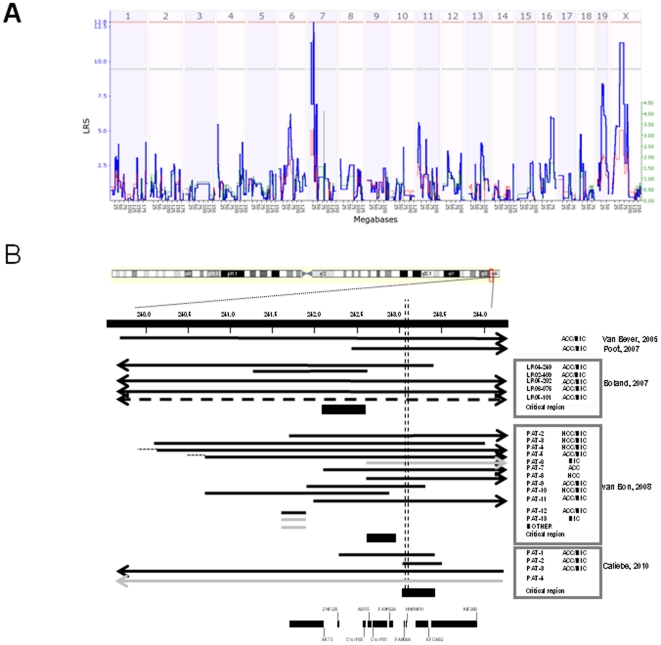
Genetic mapping of corpus callosum volume in the BXD mouse genetic reference panel. Likelihood ratio (LRS; ordinate) of the association or linkage between differences in corpus callosum volume and natural genetic variation as a function of position in the mouse genome (chromosome number and coordinates in megabses given; abscissa). Note the single genome-wide significant peak in mouse chromosome 7 (A). Overlapping deletions in genomic region 1q44 in patients with an ACC (B). Black bars and arrows indicate hemizygously deleted regions. Grey bars and arrows indicate hemizygous deletions found in subject with a normal corpus callosum. Vertical lines indicate the region that is hemizygous in 32 out of 41 cases, and overlaps with the mouse QTL.

Here, we focused our analysis on the corpus callosum volume spanning a wide range (∼8.5 to 17.6 mm^3^) across individuals of the BXD panel (with known genotypes). Through interval mapping (using the online GeneNetwork system at www.genenetwork.org) we identified a single significant QTL on mouse chromosome 7 for corpus callosum volume with a QTL peak located between approximately 25.5 and 26.7 Mb (**Figure 1A**). The peak of the QTL-interval just reached genome-wide significance level with a log of odds (LOD) score of 2.8 (permutation likelihood ratio statistic, or LRS, threshold is computed of the genome-wide p value of 0.05 using 1000 permutations). This locus on chromosome 7 contains approximately 46 genes.

Comparing the genes in this QTL region with those associated with syndromes involving an ACC (**Table 1**) and those covered by CNVs yielded a single gene overlap, namely the *HNRPU* gene in humans and its homolog *Hnrpul1* in mice. Further analysis in BXD strains revealed that the corpus callosum was significantly larger in strains with a *B* genotype at the *Hnrpul1* locus (15.53±1.90 mm^3^) compared to strains with a *D* genotype (10.85±2.65 mm^3^) (ANOVA analysis: F = 22.48, p<9.87*10^−5^)). The gene encoding *HNRPU* is located in the q44 region of human chromosome 1 and is included in the hemizygous 1q44 losses of 32 out of the 41 published cases showing an ACC on MRI (**Figure 1B**). Taken together, these suggest that humans and mice share a single locus encompassing one gene that may control corpus callosum development. Interestingly, the mouse locus also contains *Zfp260* and *Dmpk*, two highly polymorphic genes associated with cis eQTL in the neocortex (data not shown), suggesting that there may also be a small complex of genes that modulate neocortical development in close proximity on chromosome 7 in mouse. Under the proviso that an ACC is the outcome of a perturbation of neurodevelopment, it is conceivable that proper development of the corpus callosum is controlled by a gene involved in the processing of primary transcripts in the nucleus, which functions in the mouse as a quantitative trait locus.

## Discussion

Here we have presented an approach to identify genes that may control brain development associated with human disease. For this, we made use of evolutionary conservation of the development of the corpus callosum in relation to natural genetic variation. Thus, mapping of loci for an inherited defect in human patients is complemented by experimental data generated by crossing inbred mouse strains. Such crosses of inbred mice strains provide a tool for “genetic experiments” to test such hypotheses and select relevant human candidate genes from the many genes currently associated with syndromes involving an ACC. We hypothesize that comparing patterns of genetic control of conserved developmental processes among animal species may be fruitful for other, not only neurological, developmental processes. Very recent studies indicated that cross-species genome comparisons in relation to preserved phenotypes may also apply to other complex disorders, such as hypertension [32] and psychiatric disorders [33], and thus may open new roads for understanding disease etiology.

This conjecture prompts several ramifications. First, the development of the corpus callosum may be under the control of a transcriptional network composed of *HNRPU* and its putative downstream targets. Second, hemizygous losses of *HNRPU* may not be the sole genetic cause of an ACC. Several studies have demonstrated that hemizygosity for *HNRPU* is not sufficient to cause an ACC [14], [15], [34]. However, any mutation affecting proper functioning of the *HNRPU* gene product and its interaction with other proteins may lead to an ACC. These mutations are not necessarily limited to the *HNRPU* gene product, but may include genes encoding downstream targets. Such genes may either have been identified in syndromes involving an ACC or may be covered by the CNVs found in sporadic patients with an ACC, or have as yet not been identified. This offers a potential explanation for the phenotypic diversity of patients with hemizygous losses in 1q44. It also suggests an explanation for the heterogeneity of loci and genes potentially involving an ACC. Given the clinical importance of an ACC, and the many ramifications of this hypothesis, functional experimental tests appear worthwhile. Furthermore, this approach of integrating mouse genetic mapping data of evolutionary conserved phenotypes may also proof useful for a wide variety of complex human diseases, such as congenital heart disease, eating disorders, and autism spectrum disorders.

## Materials and Methods

### Prioritizing candidate loci and genes

To systematically determine genes or CNVs that were specifically found among patients with an ACC, or in syndromes involving an ACC, we analyzed our data using the Gene Annotation Tool to Help Explain Relationships (GATHER) developed by Chang and Nevins [12]. The algorithms embedded herein allow to determine significance of association with regards to shared biological processes (using Gene Ontology: http://www.geneontology.org/GO.doc.shtml), chromosomal locations or biochemical pathways (using KEGG: http://www.genome.jp/kegg/). The algorithms generate p values (assuming a normal distribution of likelihoods) and a Bayes factor, indicating the fold-likelihood that a model fits the data vs. the neutral null-hypothesis, to indicate significance of association of GO categories with an ACC [12].

### Gene mapping with crosses of inbred mouse strains

To study genetic factors that contribute to differences in brain structure we focused on a subset of fully inbred BXD RI strains, where each of the strains contains a unique genetic pattern of the genomes from the maternal and paternal strains. Age-matched pairs (male and female) of mice belonging to 11 inbred strains (56–64 days of age) were obtained directly from the Jackson Laboratory (www.jax.org): C57BL/6J (B6), DBA/2J (D2), and the following nine BXD recombinant inbred strains—BXD1, BXD6, BXD15, BXD16, BXD24, BXD28, BXD29, BXD34, and BXD40. We intentionally studied age-matched male–female pairs from different litters. To ensure that the low levels of within-strain variance are not simply due to a common litter effects, we chose that same strain mice coming from different litters. (This is analogous to the situation of monozygotic human twins raised in different environments.)

To study structural variation of the brain in this family of strains we used high-resolution magnetic resonance microscopy (MRM) imaging techniques [35] of actively stained brain specimens, followed by semi-automated segmentation of the brain images [36]–[39] into 33 major regions — gray matter nuclei, white matter fibers, and ventricular space.

Imaging was performed at the Duke Center for In Vivo Microscopy. All experiments were conducted in accordance with NIH guidelines, using protocols approved by the Duke University Institutional Animal Care and Use Review Committee under IACUC protocol number A123-07-04. The Duke animal program has AAALAC accreditation number 363, since 1976; NIH/PHS assurance number A3195-01, current through 2013. Mice were anesthetized with 100 mg/kg pentobarbital (i.p.) and then fixed by transcardial perfusion, first with a flush of a mixture of 0.9% saline and gadoteridol contrast agent —ProHance (Bracco Diagnostics, Princeton, NJ) (10∶1, v∶v), followed by a mixture of 10% formalin and ProHance (10∶1, v∶v). Whole heads were stored overnight in formalin, and then trimmed to remove the lower jaw and muscle. Brains were scanned within the cranial vault to avoid distortions or damage to the tissue during excision from the cranium. The fixed specimens were imaged using a 9.4 T (400 MHz) vertical bore Oxford magnet with a GE EXCITE console (Epic 11.0). A 14-mm diameter solenoid RF coil was used for the ex-vivo, in-situ mouse brains. We used a 3D spin warp sequence with the readout gradient applied along the long (anterior–posterior) axis of the brain. The multispectral data consist of a T1- and a T2-weighted imaging protocols. The T1-weighted sequence was acquired with an echo time (TE) of 5.1 ms, repetition time (TR) 50 ms, 62.5 kHz bandwidth, field of view (FOV) of 11×11×22 mm. A T2 multiecho sequence was acquired with a Carr Purcell Meiboom Gill sequence using the same FOV and bandwidth, with TR of 400 ms and echo spacing of 7 ms (16 echoes). To produce data heavily dependent on T2 differences the 16 echoes were MEFIC processed, i.e. Fourier transformed along the echo time line [40]. Asymmetric sampling of *k*-space with dynamic adjustment of receiver gain, and partial zero filling of *k*-space were used to achieve an image matrix size of 1024×512×512, resulting in an isotropic 21.5 µm resolution, in 2 h 7 min for the T1-weighted dataset. A matrix of 512×512×256 with isotropic resolution of 43 µm was generated for the T2-weighted data with total acquisition time of 4 h, 20 min.

We used the T1-weighted images and an atlas of the C57BL/6 mouse brain [36] as a basis for an atlas based segmentation. The T1 images were downsampled to 43 microns in order to decrease memory and computational demands. IRTK [41] was used to perform a suite of affine and nonrigid (free-form) registration between the atlas and the query datasets. The atlas labels were subjected to the same transformation that maximizes the normalized mutual information among the atlas and query images provided label sets for each query image. The resulting labels where manually corrected where necessary, using both the T1 and the T2 weighted scans, to improve the results based on local alignment.

The volumes of the segmented brain regions were calculated using MATLAB (MathWorks, Natick, MA). Strain averages for volumes of 35 independent and compound regions were entered into GeneNetwork (GN) (www.genenetwork.org). Exiting software tools within GN, including web QTL [42] were used for mapping of quantitative trait loci (QTLs) contributing to corpus callosum volume. Since only two genotypes can be investigated in these strains (B/B and D/D), all effects are analyzed under an additive model.
